# Crystal structure of 2-methyl­piperazine-1,4-diium bis­(hydrogen maleate)

**DOI:** 10.1107/S2056989015003102

**Published:** 2015-02-21

**Authors:** Intissar Wecharine, Arto Valkonen, Mohamed Rzaigui, Wajda Smirani Sta

**Affiliations:** a Laboratoire de chimie des Matériaux, Faculté des Sciences de Bizerte, Université de Carthage, 7021 Zarzouna Bizerte, Tunisia; b Department of Chemistry and Bioengineering, Tampere University of Technology, PO Box 541, 33101 Tampere, Finland

**Keywords:** crystal structure, 2-methyl­piperazine-1,4-diium, hydrogen maleate, hydrogen bonding

## Abstract

In the title salt, C_5_H_14_N_2_^2+^·2C_4_H_3_O_4_^−^, the asymmetric unit contains two independent 2-methyl­piperazinium dications, which comprise a racemic pair, and four hydrogen maleate monoanions. In the roughly planar hydrogen maleate anions, intra­molecular O—H⋯O hydrogen bonds generate *S*(7) rings. In the crystal, the four independent anions are linked to the 2-methyl­piperazinium cations through N—H⋯O hydrogen bonds, forming two-dimensional layered structures lying parallel to (001).

## Related literature   

For maleate geometry and its *S*(7) ring formation, see: Anitha *et al.* (2012 ▸). For background on 2-methyl­piperazine salts, see: Hajlaoui *et al.* (2011 ▸); Wilkinson & Harrison (2007 ▸). For a similar structure, see: Mathlouthi *et al.* (2014 ▸). For puckering parameters, see: Cremer & Pople (1975 ▸).
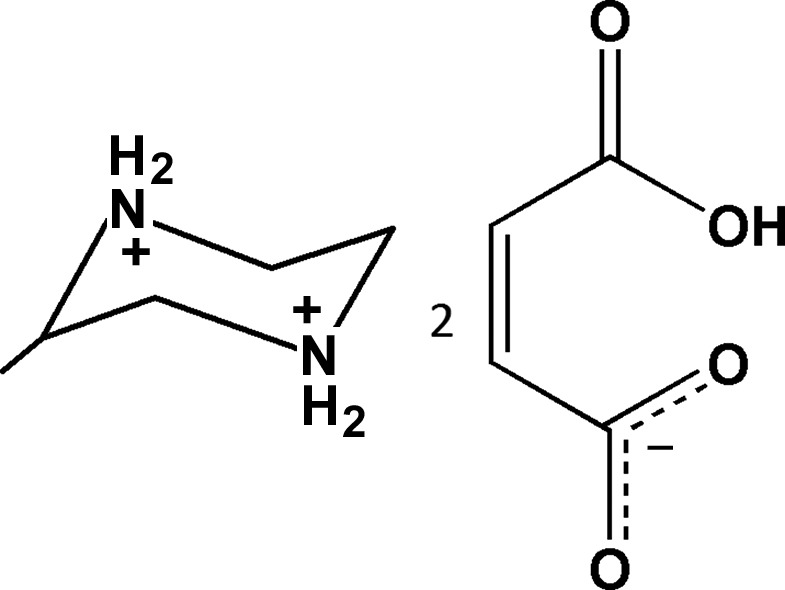


## Experimental   

### Crystal data  

C_5_H_14_N_2_^2+^·2C_4_H_3_O_4_^−^
*M* *_r_* = 332.31
Triclinic, 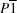

*a* = 11.4678 (9) Å
*b* = 11.4919 (9) Å
*c* = 13.3404 (13) Å
α = 71.692 (8)°
β = 75.572 (8)°
γ = 74.303 (7)°
*V* = 1580.7 (2) Å^3^
*Z* = 4
Mo *K*α radiation
μ = 0.12 mm^−1^
*T* = 170 K
0.33 × 0.14 × 0.07 mm

### Data collection  

Agilent SuperNova (single source at offset, Eos) diffractometer
Absorption correction: multi-scan (*CrysAlis PRO*; Agilent, 2013 ▸) *T*_min_ = 0.970, *T*_max_ = 0.990
10102 measured reflections
6990 independent reflections
5127 reflections with *I* > 2σ(*I*)
*R*_int_ = 0.020

### Refinement  

*R*[*F* ^2^ > 2σ(*F* ^2^)] = 0.049
*wR*(*F* ^2^) = 0.133
*S* = 1.06
6990 reflections
451 parameters
8 restraints
H atoms treated by a mixture of independent and constrained refinement
Δρ_max_ = 0.27 e Å^−3^
Δρ_min_ = −0.23 e Å^−3^

### 

Data collection: *CrysAlis PRO* (Agilent, 2013 ▸); cell refinement: *CrysAlis PRO*; data reduction: *CrysAlis PRO*; program(s) used to solve structure: *SIR2011* (Burla *et al.*, 2012 ▸); program(s) used to refine structure: *SHELXL2013* (Sheldrick, 2008 ▸, 2015 ▸) within *WinGX* (Farrugia, 2012 ▸); molecular graphics: *ORTEP-3 for Windows* (Farrugia, 2012 ▸); software used to prepare material for publication: *SHELXL2013*.

## Supplementary Material

Crystal structure: contains datablock(s) I, General. DOI: 10.1107/S2056989015003102/zs2325sup1.cif

Structure factors: contains datablock(s) I. DOI: 10.1107/S2056989015003102/zs2325Isup2.hkl

Supporting information file. DOI: 10.1107/S2056989015003102/zs2325Isup3.cml

**Fig. 1.**
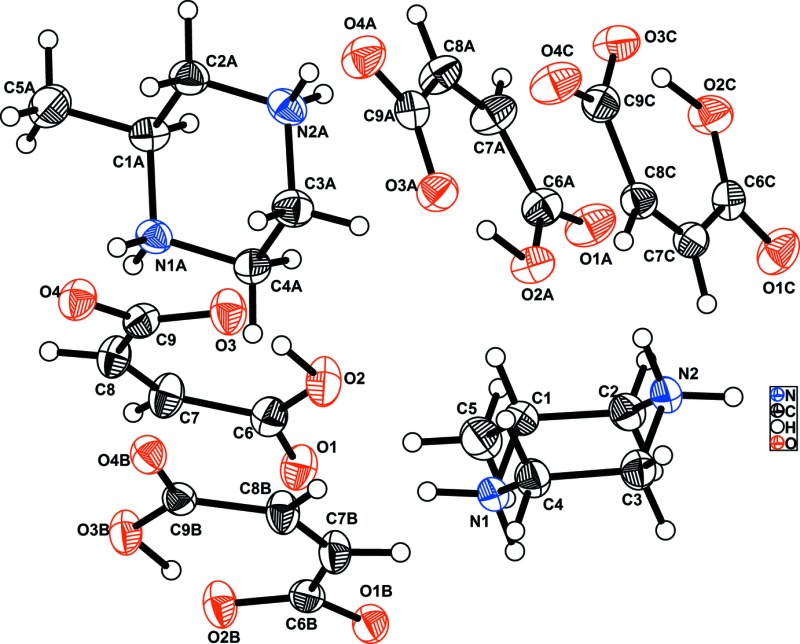
The two dications and the four anions in the asymmetric unit of the title salt, with atom numbering scheme. Displacement ellipsoids for non-H atoms are drawn at the 30% probability level.

**Fig. 2.**
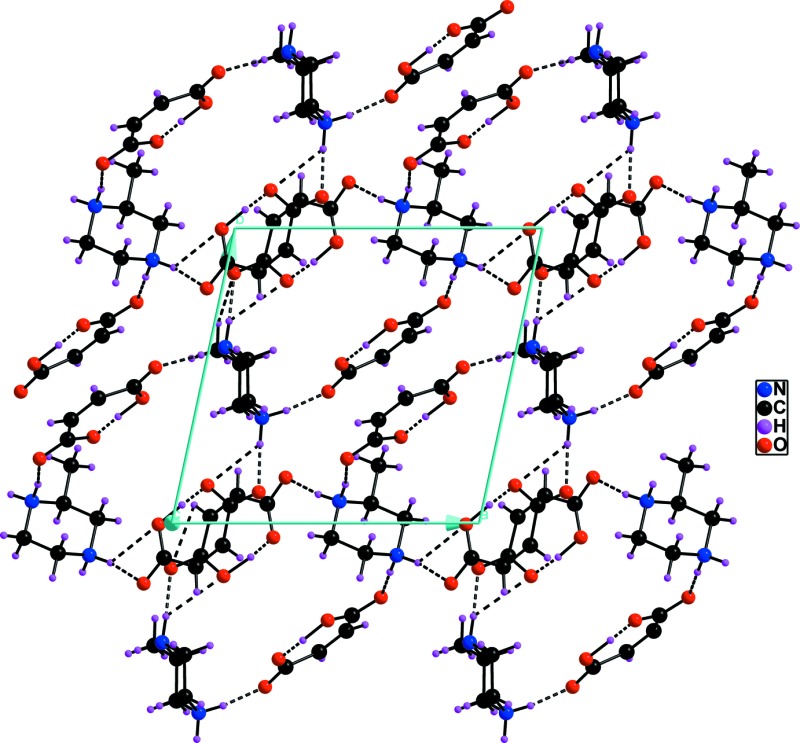
A view of a layerered structure of the title compound along the c axis showing the two-dimensional layers lying parallel to the (001) plane. Hydrogen bonds are denoted by dashed lines.

**Fig. 3.**
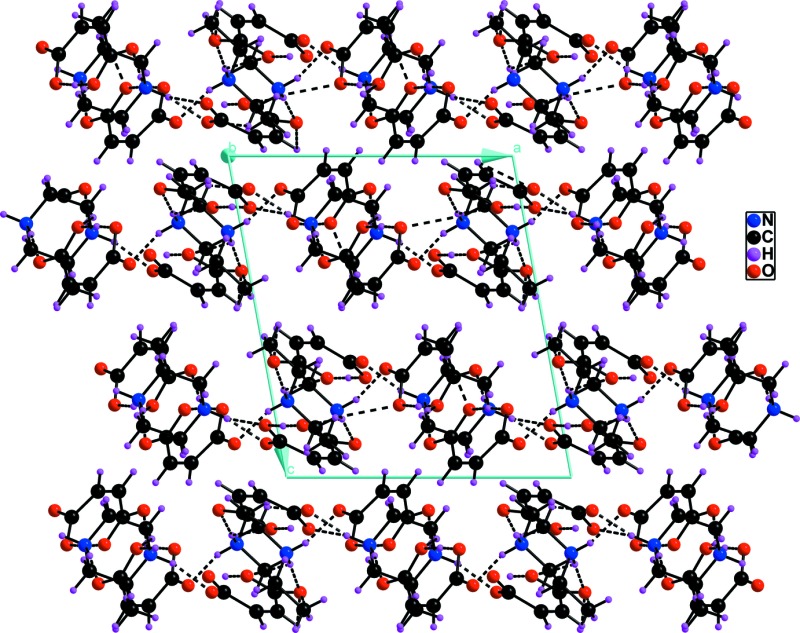
The structure of the title compound viewed along the b axis.

. DOI: 10.1107/S2056989015003102/zs2325fig1.tif
The two dications and the four anions in the asymmetric unit of the title salt, with atom numbering scheme. Displacement ellipsoids for non-H atoms are drawn at the 30% probability level.

c . DOI: 10.1107/S2056989015003102/zs2325fig2.tif
A view of a layerered structure of the title compound along the *c* axis showing the two-dimensional layers lying parallel to the (001) plane. Hydrogen bonds are denoted by dashed lines.

b . DOI: 10.1107/S2056989015003102/zs2325fig3.tif
The structure of the title compound viewed along the *b* axis.

CCDC reference: 1049285

Additional supporting information:  crystallographic information; 3D view; checkCIF report

## Figures and Tables

**Table 1:** Hydrogen-bond geometry (, )

*D*H*A*	*D*H	H*A*	*D* *A*	*D*H*A*
N1H1*C*O4*C* ^i^	0.93(1)	1.91(2)	2.802(2)	162(2)
N1H1*D*O1	0.93(2)	1.89(2)	2.801(2)	166(2)
N2H2*E*O1*A*	0.94(2)	1.80(2)	2.723(2)	168(2)
N2H2*F*O3*B* ^ii^	0.95(2)	2.50(2)	3.187(2)	129(2)
N2H2*F*O4*B* ^ii^	0.95(2)	1.82(2)	2.760(2)	169(2)
N1*A*H1*E*O3	0.96(1)	2.59(2)	3.279(2)	129(2)
N1*A*H1*E*O4	0.96(1)	1.86(2)	2.811(2)	169(2)
N1*A*H1*F*O1*C* ^iii^	0.92(2)	1.90(2)	2.787(2)	162(2)
N2*A*H2*G*O4*A*	0.92(2)	1.81(2)	2.710(2)	164(2)
N2*A*H2*H*O1*B* ^iv^	0.95(2)	1.82(2)	2.764(2)	168(2)
N2*A*H2*H*O2*B* ^iv^	0.95(2)	2.51(2)	3.191(2)	128(2)
O2H2O3	1.15(3)	1.28(3)	2.4258(19)	174(2)
O2*A*H2*I*O3*A*	1.21(2)	1.22(2)	2.4240(19)	175(2)
O3*B*H2*J*O2*B*	1.18(2)	1.24(2)	2.4174(18)	177(2)
O2*C*H2*K*O3*C*	1.20(2)	1.22(2)	2.4192(19)	174(2)

Symmetry codes: (i) 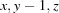; (ii) 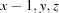; (iii) 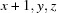; (iv) 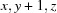.

## supplementary crystallographic information

### S1. Comment

Our ongoing studies of novel salts of maleic acid with related substances arises from the fact that hydrogen maleate anions in these systems possess short but highly strained hydrogen bonds in salts with racemic amines (Hajlaoui *et al.*, 2011; Wilkinson & Harrison, 2007). We report herein the synthesis and structure of the title hydrogen maleate salt with 2-methylpiperazine, (C_5_ H_14_ N_2_)^2+^ 2(C_4_H_3_O_4_^-^).

As shown in Fig. 1, the asymmetric unit of the title salt contains two independent 2-methylpipirazinium dications which form a racemic pair [C1(*R*) and C1*A*(*S*)] and four hydrogen maleate anions. In the planar hydrogen maleate anions, short intramolecular O—H···O hydrogen bonds (Table 1) generate *S*(7) rings. This is common in many structures of maleic acid as the *cis* disposition of the alkene places hydrogen-bonding donors and acceptors in close proximity (Mathlouthi *et al.*, 2014).

In the crystal (Fig. 2), the piperazinium groups of the cation are hydrogen-bonded to the carboxylate O atoms of the anion *via* N—H···O hydrogen bonds, forming a two-dimensional network. The four maleate anions (C6–C9), (C6*A*–C9*A*), (C6*B*–C9*B*) and (C6*C*–C9*C*) are connected to the organic cations, forming two-dimensional layers lying parallel to (001) (Fig. 3).

In the cation, the piperazinium rings adopt distorted chair conformations [puckering parameters Q, *θ*, and *φ* = 0.574 (2) Å, 1.11 (1)° and 73.6 (1)° for the first cation and = 0.577 (2) Å, 1.83 (2)° and 73.83 (1) for the second] (Cremer & Pople, 1975).

### S2. Experimental

A mixture of maleic acid (1*M*) and 2-methylpiperazine dissolved in ethanol (molar ratio 1:1:1) was stirred for 2 h and then kept at room temperature. Transparent crystals of the title compound were obtained one week later.

### S3. Refinement

All H atoms bonded to C atoms of organic cations were positioned geometrically and treated as riding on their parent atoms, [C—H = 0.99 Å] with *U*_iso_(H) = 1.2 *U*_eq_(C). H-atoms attached to O and N atoms were located in difference Fourier maps and the positional paramaters for those attached to O were refined, with *U*_iso_(H) = 1.5 *U*_eq_(O) while the N—H bond distances were allowed to ride with N—H restrained at 0.91 (2) Å and *U*_iso_(H) = 1.2 *U*_eq_(N).

### Crystal data

**Table:**

C_5_H_14_N_2_^2+^·2C_4_H_3_O_4_^−^	*Z* = 4
*M**_r_* = 332.31	*F*(000) = 704
Triclinic, *P*1	*D*_x_ = 1.396 Mg m^−^^3^
*a* = 11.4678 (9) Å	Mo *K*α radiation, λ = 0.71073 Å
*b* = 11.4919 (9) Å	Cell parameters from 3309 reflections
*c* = 13.3404 (13) Å	θ = 2.2–28.5°
α = 71.692 (8)°	µ = 0.12 mm^−^^1^
β = 75.572 (8)°	*T* = 170 K
γ = 74.303 (7)°	Prism, colourless
*V* = 1580.7 (2) Å^3^	0.33 × 0.14 × 0.07 mm

### Data collection

**Table:**

Agilent SuperNova (single source at offset, Eos) diffractometer	6990 independent reflections
Mirror monochromator	5127 reflections with *I* > 2σ(*I*)
Detector resolution: 16.0107 pixels mm^-1^	*R*_int_ = 0.020
ω scans	θ_max_ = 29.0°, θ_min_ = 1.9°
Absorption correction: multi-scan (*CrysAlis PRO*; Agilent, 2013)	*h* = −14→14
*T*_min_ = 0.970, *T*_max_ = 0.990	*k* = −15→11
10102 measured reflections	*l* = −17→18

### Refinement

**Table:**

Refinement on *F*^2^	8 restraints
Least-squares matrix: full	Hydrogen site location: mixed
*R*[*F*^2^ > 2σ(*F*^2^)] = 0.049	H atoms treated by a mixture of independent and constrained refinement
*wR*(*F*^2^) = 0.133	*w* = 1/[σ^2^(*F*_o_^2^) + (0.0497*P*)^2^ + 0.2961*P*] where *P* = (*F*_o_^2^ + 2*F*_c_^2^)/3
*S* = 1.06	(Δ/σ)_max_ < 0.001
6990 reflections	Δρ_max_ = 0.27 e Å^−^^3^
451 parameters	Δρ_min_ = −0.23 e Å^−^^3^

### Special details

**Table:**

Geometry. All e.s.d.'s (except the e.s.d. in the dihedral angle between two l.s. planes) are estimated using the full covariance matrix. The cell e.s.d.'s are taken into account individually in the estimation of e.s.d.'s in distances, angles and torsion angles; correlations between e.s.d.'s in cell parameters are only used when they are defined by crystal symmetry. An approximate (isotropic) treatment of cell e.s.d.'s is used for estimating e.s.d.'s involving l.s. planes.

### Fractional atomic coordinates and isotropic or equivalent isotropic displacement parameters (Å^2^)

**Table:**

	*x*	*y*	*z*	*U*_iso_*/*U*_eq_	
N1	0.47112 (14)	−0.07313 (15)	0.24023 (13)	0.0264 (3)	
H1C	0.4636 (18)	−0.1306 (16)	0.2079 (15)	0.032*	
H1D	0.5432 (16)	−0.0963 (18)	0.2683 (15)	0.032*	
N2	0.24684 (15)	0.10974 (15)	0.21164 (13)	0.0289 (4)	
H2E	0.2571 (19)	0.1670 (17)	0.2438 (15)	0.035*	
H2F	0.1734 (16)	0.1357 (18)	0.1822 (15)	0.035*	
C1	0.36652 (17)	−0.06564 (18)	0.33304 (15)	0.0285 (4)	
H1	0.3764	−0.0068	0.3704	0.034*	
C2	0.24641 (17)	−0.01499 (18)	0.29123 (16)	0.0303 (4)	
H2A	0.2339	−0.0745	0.2569	0.036*	
H2B	0.1773	−0.0068	0.3519	0.036*	
C3	0.35045 (17)	0.10233 (19)	0.11976 (16)	0.0317 (4)	
H3A	0.3505	0.1868	0.0698	0.038*	
H3B	0.3398	0.0471	0.0803	0.038*	
C4	0.47168 (17)	0.05140 (18)	0.15914 (16)	0.0306 (4)	
H4A	0.5394	0.0420	0.0977	0.037*	
H4B	0.4863	0.1112	0.1919	0.037*	
C5	0.3686 (2)	−0.19420 (19)	0.41174 (17)	0.0389 (5)	
H5A	0.4474	−0.2245	0.4372	0.058*	
H5B	0.3588	−0.2526	0.3759	0.058*	
H5C	0.3011	−0.1885	0.4729	0.058*	
N1A	0.94928 (15)	0.40524 (14)	0.23662 (13)	0.0261 (3)	
H1E	0.9556 (18)	0.3171 (14)	0.2709 (15)	0.031*	
H1F	1.0225 (15)	0.4310 (18)	0.2052 (15)	0.031*	
N2A	0.78144 (15)	0.64376 (15)	0.20796 (14)	0.0290 (4)	
H2G	0.7075 (15)	0.6198 (19)	0.2407 (16)	0.035*	
H2H	0.7684 (19)	0.7327 (14)	0.1807 (15)	0.035*	
C1A	0.89052 (18)	0.46035 (17)	0.32972 (15)	0.0294 (4)	
H1A	0.8112	0.4321	0.3635	0.035*	
C2A	0.86258 (18)	0.60249 (17)	0.28913 (16)	0.0306 (4)	
H2C	0.8216	0.6396	0.3500	0.037*	
H2D	0.9404	0.6322	0.2567	0.037*	
C3A	0.83774 (19)	0.58864 (17)	0.11580 (15)	0.0319 (4)	
H3C	0.9137	0.6198	0.0775	0.038*	
H3D	0.7798	0.6150	0.0650	0.038*	
C4A	0.86848 (18)	0.44718 (17)	0.15408 (15)	0.0289 (4)	
H4C	0.7916	0.4155	0.1853	0.035*	
H4D	0.9110	0.4120	0.0924	0.035*	
C5A	0.9731 (2)	0.4155 (2)	0.41267 (18)	0.0428 (5)	
H5D	0.9892	0.3237	0.4373	0.064*	
H5E	0.9325	0.4506	0.4738	0.064*	
H5F	1.0511	0.4429	0.3807	0.064*	
O1	0.66248 (13)	−0.15326 (13)	0.35791 (11)	0.0348 (3)	
O2	0.66093 (13)	0.04943 (13)	0.30938 (12)	0.0395 (4)	
H2	0.720 (2)	0.116 (2)	0.3089 (19)	0.059*	
O3	0.79597 (13)	0.18044 (12)	0.30752 (11)	0.0354 (3)	
O4	0.97246 (13)	0.15407 (12)	0.35879 (11)	0.0326 (3)	
C6	0.70396 (18)	−0.06453 (18)	0.35806 (15)	0.0296 (4)	
C7	0.80662 (18)	−0.09258 (18)	0.41811 (15)	0.0319 (4)	
H7	0.8216	−0.1751	0.4640	0.038*	
C8	0.88045 (18)	−0.02073 (18)	0.41771 (15)	0.0306 (4)	
H8	0.9404	−0.0603	0.4626	0.037*	
C9	0.88357 (18)	0.11290 (17)	0.35741 (15)	0.0276 (4)	
O1A	0.26932 (14)	0.25263 (14)	0.33171 (12)	0.0416 (4)	
O2A	0.42635 (13)	0.33042 (13)	0.22241 (11)	0.0342 (3)	
H2I	0.493 (2)	0.398 (2)	0.2194 (18)	0.051*	
O3A	0.55889 (13)	0.46177 (13)	0.22439 (11)	0.0342 (3)	
O4A	0.58356 (13)	0.55559 (14)	0.33715 (12)	0.0396 (4)	
C6A	0.35063 (19)	0.31324 (18)	0.31199 (16)	0.0311 (4)	
C7A	0.3585 (2)	0.36602 (19)	0.39852 (16)	0.0355 (5)	
H7A	0.3016	0.3453	0.4632	0.043*	
C8A	0.43201 (19)	0.43722 (19)	0.40013 (16)	0.0334 (5)	
H8A	0.4193	0.4589	0.4657	0.040*	
C9A	0.53077 (18)	0.48811 (18)	0.31542 (16)	0.0300 (4)	
O1B	0.73978 (12)	−0.10379 (12)	0.10276 (11)	0.0303 (3)	
O2B	0.91092 (12)	−0.12659 (12)	0.16097 (11)	0.0346 (3)	
H2J	0.980 (2)	−0.060 (2)	0.1591 (18)	0.052*	
O3B	1.04304 (13)	0.00525 (12)	0.16145 (11)	0.0340 (3)	
O4B	1.05050 (12)	0.20575 (12)	0.10410 (11)	0.0294 (3)	
C6B	0.82359 (17)	−0.05937 (17)	0.11055 (15)	0.0258 (4)	
C7B	0.81991 (18)	0.07654 (17)	0.05890 (16)	0.0307 (4)	
H7B	0.7552	0.1182	0.0193	0.037*	
C8B	0.89284 (18)	0.14906 (17)	0.05930 (16)	0.0307 (4)	
H8B	0.8715	0.2341	0.0201	0.037*	
C9B	1.00213 (16)	0.11871 (17)	0.11108 (14)	0.0243 (4)	
O1C	0.16316 (13)	0.47339 (14)	0.10385 (12)	0.0409 (4)	
O2C	0.18351 (13)	0.57671 (13)	0.21065 (11)	0.0367 (3)	
H2K	0.250 (2)	0.640 (2)	0.2150 (18)	0.055*	
O3C	0.31604 (13)	0.70898 (14)	0.20988 (11)	0.0373 (3)	
O4C	0.47555 (14)	0.78285 (14)	0.10389 (12)	0.0417 (4)	
C6C	0.21839 (17)	0.53961 (18)	0.12438 (15)	0.0295 (4)	
C7C	0.32705 (17)	0.57784 (17)	0.04445 (15)	0.0282 (4)	
H7C	0.3474	0.5450	−0.0162	0.034*	
C8C	0.40062 (17)	0.65074 (17)	0.04398 (15)	0.0279 (4)	
H8C	0.4646	0.6614	−0.0169	0.033*	
C9C	0.39798 (18)	0.71797 (18)	0.12411 (16)	0.0299 (4)	

### Atomic displacement parameters (Å^2^)

**Table:**

	*U*^11^	*U*^22^	*U*^33^	*U*^12^	*U*^13^	*U*^23^
N1	0.0202 (8)	0.0294 (8)	0.0316 (9)	−0.0010 (7)	−0.0068 (7)	−0.0129 (7)
N2	0.0219 (8)	0.0305 (9)	0.0368 (9)	−0.0005 (7)	−0.0096 (7)	−0.0133 (7)
C1	0.0240 (10)	0.0335 (10)	0.0285 (10)	−0.0021 (8)	−0.0043 (8)	−0.0126 (8)
C2	0.0242 (10)	0.0337 (10)	0.0330 (11)	−0.0051 (8)	−0.0030 (8)	−0.0115 (9)
C3	0.0263 (10)	0.0364 (11)	0.0326 (11)	−0.0048 (8)	−0.0070 (8)	−0.0096 (9)
C4	0.0251 (10)	0.0321 (10)	0.0349 (11)	−0.0045 (8)	−0.0061 (8)	−0.0098 (9)
C5	0.0362 (12)	0.0397 (12)	0.0357 (12)	−0.0023 (9)	−0.0057 (10)	−0.0083 (10)
N1A	0.0256 (8)	0.0214 (8)	0.0313 (9)	−0.0044 (7)	−0.0039 (7)	−0.0084 (7)
N2A	0.0267 (9)	0.0212 (8)	0.0373 (10)	−0.0047 (7)	−0.0024 (7)	−0.0082 (7)
C1A	0.0339 (11)	0.0275 (10)	0.0278 (10)	−0.0070 (8)	−0.0033 (8)	−0.0099 (8)
C2A	0.0314 (10)	0.0274 (10)	0.0347 (11)	−0.0053 (8)	−0.0043 (9)	−0.0126 (8)
C3A	0.0361 (11)	0.0268 (10)	0.0311 (11)	−0.0056 (8)	−0.0049 (9)	−0.0071 (8)
C4A	0.0304 (10)	0.0263 (10)	0.0322 (10)	−0.0046 (8)	−0.0079 (8)	−0.0102 (8)
C5A	0.0502 (14)	0.0408 (12)	0.0428 (13)	−0.0060 (10)	−0.0181 (11)	−0.0137 (10)
O1	0.0325 (8)	0.0370 (8)	0.0390 (8)	−0.0134 (6)	−0.0114 (6)	−0.0064 (6)
O2	0.0367 (8)	0.0336 (8)	0.0508 (9)	−0.0078 (7)	−0.0212 (7)	−0.0039 (7)
O3	0.0345 (8)	0.0281 (7)	0.0428 (8)	−0.0056 (6)	−0.0155 (7)	−0.0026 (6)
O4	0.0360 (8)	0.0271 (7)	0.0389 (8)	−0.0095 (6)	−0.0129 (6)	−0.0074 (6)
C6	0.0275 (10)	0.0338 (11)	0.0275 (10)	−0.0095 (8)	−0.0040 (8)	−0.0060 (8)
C7	0.0343 (11)	0.0312 (10)	0.0310 (11)	−0.0114 (9)	−0.0124 (9)	−0.0003 (8)
C8	0.0323 (11)	0.0313 (10)	0.0280 (10)	−0.0071 (8)	−0.0123 (9)	−0.0021 (8)
C9	0.0309 (10)	0.0287 (10)	0.0245 (10)	−0.0066 (8)	−0.0058 (8)	−0.0082 (8)
O1A	0.0520 (10)	0.0429 (9)	0.0386 (8)	−0.0252 (8)	−0.0034 (7)	−0.0133 (7)
O2A	0.0387 (8)	0.0358 (8)	0.0315 (8)	−0.0115 (6)	−0.0028 (6)	−0.0131 (6)
O3A	0.0295 (7)	0.0452 (8)	0.0301 (7)	−0.0117 (6)	0.0009 (6)	−0.0142 (6)
O4A	0.0345 (8)	0.0514 (9)	0.0414 (8)	−0.0197 (7)	−0.0025 (7)	−0.0184 (7)
C6A	0.0373 (11)	0.0271 (10)	0.0301 (11)	−0.0092 (9)	−0.0068 (9)	−0.0063 (8)
C7A	0.0437 (12)	0.0416 (12)	0.0248 (10)	−0.0203 (10)	−0.0005 (9)	−0.0085 (9)
C8A	0.0390 (12)	0.0409 (12)	0.0253 (10)	−0.0163 (9)	−0.0037 (9)	−0.0104 (9)
C9A	0.0263 (10)	0.0320 (10)	0.0322 (11)	−0.0040 (8)	−0.0075 (8)	−0.0092 (9)
O1B	0.0281 (7)	0.0263 (7)	0.0397 (8)	−0.0068 (6)	−0.0095 (6)	−0.0099 (6)
O2B	0.0296 (7)	0.0244 (7)	0.0501 (9)	−0.0057 (6)	−0.0167 (7)	−0.0026 (6)
O3B	0.0320 (8)	0.0244 (7)	0.0487 (9)	−0.0039 (6)	−0.0201 (7)	−0.0057 (6)
O4B	0.0255 (7)	0.0275 (7)	0.0369 (8)	−0.0070 (6)	−0.0064 (6)	−0.0091 (6)
C6B	0.0253 (10)	0.0259 (9)	0.0266 (10)	−0.0043 (7)	−0.0031 (8)	−0.0094 (8)
C7B	0.0295 (10)	0.0258 (10)	0.0372 (11)	−0.0042 (8)	−0.0155 (9)	−0.0028 (8)
C8B	0.0314 (11)	0.0222 (9)	0.0374 (11)	−0.0042 (8)	−0.0151 (9)	−0.0004 (8)
C9B	0.0216 (9)	0.0262 (9)	0.0255 (9)	−0.0046 (7)	−0.0022 (7)	−0.0091 (8)
O1C	0.0358 (8)	0.0560 (10)	0.0394 (8)	−0.0223 (7)	−0.0057 (7)	−0.0146 (7)
O2C	0.0316 (8)	0.0453 (9)	0.0355 (8)	−0.0138 (7)	0.0037 (6)	−0.0167 (7)
O3C	0.0392 (8)	0.0422 (8)	0.0353 (8)	−0.0133 (7)	0.0011 (7)	−0.0189 (7)
O4C	0.0528 (10)	0.0419 (9)	0.0391 (8)	−0.0253 (8)	−0.0023 (7)	−0.0140 (7)
C6C	0.0253 (10)	0.0316 (10)	0.0311 (10)	−0.0040 (8)	−0.0086 (8)	−0.0067 (8)
C7C	0.0287 (10)	0.0294 (10)	0.0258 (10)	−0.0027 (8)	−0.0063 (8)	−0.0079 (8)
C8C	0.0273 (10)	0.0295 (10)	0.0253 (10)	−0.0046 (8)	−0.0037 (8)	−0.0070 (8)
C9C	0.0322 (11)	0.0266 (10)	0.0314 (11)	−0.0059 (8)	−0.0075 (9)	−0.0071 (8)

### Geometric parameters (Å, º)

**Table:**

N1—C4	1.499 (2)	O2—C6	1.287 (2)
N1—C1	1.500 (2)	O2—H2	1.15 (3)
N1—H1C	0.926 (14)	O3—C9	1.281 (2)
N1—H1D	0.932 (15)	O3—H2	1.28 (3)
N2—C3	1.485 (2)	O4—C9	1.241 (2)
N2—C2	1.492 (2)	C6—C7	1.495 (3)
N2—H2E	0.936 (15)	C7—C8	1.331 (3)
N2—H2F	0.953 (15)	C7—H7	0.9500
C1—C2	1.514 (3)	C8—C9	1.499 (3)
C1—C5	1.518 (3)	C8—H8	0.9500
C1—H1	1.0000	O1A—C6A	1.240 (2)
C2—H2A	0.9900	O2A—C6A	1.284 (2)
C2—H2B	0.9900	O2A—H2I	1.21 (2)
C3—C4	1.510 (3)	O3A—C9A	1.286 (2)
C3—H3A	0.9900	O3A—H2I	1.22 (2)
C3—H3B	0.9900	O4A—C9A	1.239 (2)
C4—H4A	0.9900	C6A—C7A	1.493 (3)
C4—H4B	0.9900	C7A—C8A	1.332 (3)
C5—H5A	0.9800	C7A—H7A	0.9500
C5—H5B	0.9800	C8A—C9A	1.486 (3)
C5—H5C	0.9800	C8A—H8A	0.9500
N1A—C4A	1.496 (2)	O1B—C6B	1.242 (2)
N1A—C1A	1.498 (2)	O2B—C6B	1.282 (2)
N1A—H1E	0.962 (14)	O2B—H2J	1.24 (2)
N1A—H1F	0.921 (15)	O3B—C9B	1.284 (2)
N2A—C3A	1.484 (2)	O3B—H2J	1.18 (2)
N2A—C2A	1.485 (2)	O4B—C9B	1.238 (2)
N2A—H2G	0.923 (15)	C6B—C7B	1.490 (3)
N2A—H2H	0.954 (15)	C7B—C8B	1.333 (2)
C1A—C5A	1.512 (3)	C7B—H7B	0.9500
C1A—C2A	1.520 (3)	C8B—C9B	1.488 (3)
C1A—H1A	1.0000	C8B—H8B	0.9500
C2A—H2C	0.9900	O1C—C6C	1.238 (2)
C2A—H2D	0.9900	O2C—C6C	1.286 (2)
C3A—C4A	1.512 (3)	O2C—H2K	1.20 (2)
C3A—H3C	0.9900	O3C—C9C	1.283 (2)
C3A—H3D	0.9900	O3C—H2K	1.22 (2)
C4A—H4C	0.9900	O4C—C9C	1.238 (2)
C4A—H4D	0.9900	C6C—C7C	1.491 (3)
C5A—H5D	0.9800	C7C—C8C	1.338 (2)
C5A—H5E	0.9800	C7C—H7C	0.9500
C5A—H5F	0.9800	C8C—C9C	1.493 (3)
O1—C6	1.237 (2)	C8C—H8C	0.9500
			
C4—N1—C1	111.81 (14)	C4A—C3A—H3D	109.6
C4—N1—H1C	109.4 (12)	H3C—C3A—H3D	108.1
C1—N1—H1C	108.5 (13)	N1A—C4A—C3A	110.55 (15)
C4—N1—H1D	107.7 (12)	N1A—C4A—H4C	109.5
C1—N1—H1D	106.4 (12)	C3A—C4A—H4C	109.5
H1C—N1—H1D	113.1 (17)	N1A—C4A—H4D	109.5
C3—N2—C2	111.34 (15)	C3A—C4A—H4D	109.5
C3—N2—H2E	107.0 (13)	H4C—C4A—H4D	108.1
C2—N2—H2E	110.0 (12)	C1A—C5A—H5D	109.5
C3—N2—H2F	106.1 (12)	C1A—C5A—H5E	109.5
C2—N2—H2F	108.9 (12)	H5D—C5A—H5E	109.5
H2E—N2—H2F	113.4 (17)	C1A—C5A—H5F	109.5
N1—C1—C2	109.12 (15)	H5D—C5A—H5F	109.5
N1—C1—C5	109.79 (15)	H5E—C5A—H5F	109.5
C2—C1—C5	111.37 (16)	C6—O2—H2	111.4 (12)
N1—C1—H1	108.8	C9—O3—H2	111.6 (11)
C2—C1—H1	108.8	O1—C6—O2	122.17 (17)
C5—C1—H1	108.8	O1—C6—C7	118.00 (17)
N2—C2—C1	110.87 (16)	O2—C6—C7	119.83 (17)
N2—C2—H2A	109.5	C8—C7—C6	130.47 (18)
C1—C2—H2A	109.5	C8—C7—H7	114.8
N2—C2—H2B	109.5	C6—C7—H7	114.8
C1—C2—H2B	109.5	C7—C8—C9	129.97 (18)
H2A—C2—H2B	108.1	C7—C8—H8	115.0
N2—C3—C4	110.34 (15)	C9—C8—H8	115.0
N2—C3—H3A	109.6	O4—C9—O3	122.40 (17)
C4—C3—H3A	109.6	O4—C9—C8	117.61 (17)
N2—C3—H3B	109.6	O3—C9—C8	119.99 (17)
C4—C3—H3B	109.6	C6A—O2A—H2I	111.0 (11)
H3A—C3—H3B	108.1	C9A—O3A—H2I	111.4 (11)
N1—C4—C3	110.65 (16)	O1A—C6A—O2A	122.80 (18)
N1—C4—H4A	109.5	O1A—C6A—C7A	116.90 (18)
C3—C4—H4A	109.5	O2A—C6A—C7A	120.29 (18)
N1—C4—H4B	109.5	C8A—C7A—C6A	130.79 (19)
C3—C4—H4B	109.5	C8A—C7A—H7A	114.6
H4A—C4—H4B	108.1	C6A—C7A—H7A	114.6
C1—C5—H5A	109.5	C7A—C8A—C9A	130.13 (18)
C1—C5—H5B	109.5	C7A—C8A—H8A	114.9
H5A—C5—H5B	109.5	C9A—C8A—H8A	114.9
C1—C5—H5C	109.5	O4A—C9A—O3A	122.54 (19)
H5A—C5—H5C	109.5	O4A—C9A—C8A	116.96 (17)
H5B—C5—H5C	109.5	O3A—C9A—C8A	120.50 (17)
C4A—N1A—C1A	111.40 (14)	C6B—O2B—H2J	109.4 (11)
C4A—N1A—H1E	110.4 (12)	C9B—O3B—H2J	109.6 (11)
C1A—N1A—H1E	101.4 (12)	O1B—C6B—O2B	122.06 (17)
C4A—N1A—H1F	108.5 (12)	O1B—C6B—C7B	117.68 (16)
C1A—N1A—H1F	108.9 (12)	O2B—C6B—C7B	120.26 (16)
H1E—N1A—H1F	116.1 (18)	C8B—C7B—C6B	130.34 (17)
C3A—N2A—C2A	111.72 (15)	C8B—C7B—H7B	114.8
C3A—N2A—H2G	109.8 (13)	C6B—C7B—H7B	114.8
C2A—N2A—H2G	108.4 (13)	C7B—C8B—C9B	130.62 (17)
C3A—N2A—H2H	107.6 (12)	C7B—C8B—H8B	114.7
C2A—N2A—H2H	109.1 (12)	C9B—C8B—H8B	114.7
H2G—N2A—H2H	110.3 (18)	O4B—C9B—O3B	122.09 (16)
N1A—C1A—C5A	110.46 (16)	O4B—C9B—C8B	117.92 (16)
N1A—C1A—C2A	109.13 (15)	O3B—C9B—C8B	119.99 (16)
C5A—C1A—C2A	111.50 (16)	C6C—O2C—H2K	111.3 (11)
N1A—C1A—H1A	108.6	C9C—O3C—H2K	111.7 (11)
C5A—C1A—H1A	108.6	O1C—C6C—O2C	121.98 (18)
C2A—C1A—H1A	108.6	O1C—C6C—C7C	117.87 (17)
N2A—C2A—C1A	110.29 (15)	O2C—C6C—C7C	120.14 (17)
N2A—C2A—H2C	109.6	C8C—C7C—C6C	130.72 (18)
C1A—C2A—H2C	109.6	C8C—C7C—H7C	114.6
N2A—C2A—H2D	109.6	C6C—C7C—H7C	114.6
C1A—C2A—H2D	109.6	C7C—C8C—C9C	130.18 (18)
H2C—C2A—H2D	108.1	C7C—C8C—H8C	114.9
N2A—C3A—C4A	110.50 (15)	C9C—C8C—H8C	114.9
N2A—C3A—H3C	109.6	O4C—C9C—O3C	122.04 (18)
C4A—C3A—H3C	109.6	O4C—C9C—C8C	117.88 (18)
N2A—C3A—H3D	109.6	O3C—C9C—C8C	120.08 (17)
			
C4—N1—C1—C2	56.7 (2)	C6—C7—C8—C9	0.8 (4)
C4—N1—C1—C5	179.01 (15)	C7—C8—C9—O4	−170.0 (2)
C3—N2—C2—C1	58.3 (2)	C7—C8—C9—O3	10.4 (3)
N1—C1—C2—N2	−57.02 (19)	O1A—C6A—C7A—C8A	−177.6 (2)
C5—C1—C2—N2	−178.38 (15)	O2A—C6A—C7A—C8A	2.9 (3)
C2—N2—C3—C4	−57.0 (2)	C6A—C7A—C8A—C9A	0.2 (4)
C1—N1—C4—C3	−56.6 (2)	C7A—C8A—C9A—O4A	178.0 (2)
N2—C3—C4—N1	55.7 (2)	C7A—C8A—C9A—O3A	−2.2 (3)
C4A—N1A—C1A—C5A	179.32 (16)	O1B—C6B—C7B—C8B	176.1 (2)
C4A—N1A—C1A—C2A	−57.8 (2)	O2B—C6B—C7B—C8B	−3.9 (3)
C3A—N2A—C2A—C1A	−58.2 (2)	C6B—C7B—C8B—C9B	0.1 (4)
N1A—C1A—C2A—N2A	57.7 (2)	C7B—C8B—C9B—O4B	−176.0 (2)
C5A—C1A—C2A—N2A	−179.99 (16)	C7B—C8B—C9B—O3B	3.6 (3)
C2A—N2A—C3A—C4A	56.7 (2)	O1C—C6C—C7C—C8C	177.83 (19)
C1A—N1A—C4A—C3A	57.0 (2)	O2C—C6C—C7C—C8C	−1.2 (3)
N2A—C3A—C4A—N1A	−55.4 (2)	C6C—C7C—C8C—C9C	0.1 (3)
O1—C6—C7—C8	167.9 (2)	C7C—C8C—C9C—O4C	−178.34 (19)
O2—C6—C7—C8	−12.7 (3)	C7C—C8C—C9C—O3C	0.7 (3)

### Hydrogen-bond geometry (Å, º)

**Table:**

*D*—H···*A*	*D*—H	H···*A*	*D*···*A*	*D*—H···*A*
N1—H1*C*···O4*C*^i^	0.93 (1)	1.91 (2)	2.802 (2)	162 (2)
N1—H1*D*···O1	0.93 (2)	1.89 (2)	2.801 (2)	166 (2)
N2—H2*E*···O1*A*	0.94 (2)	1.80 (2)	2.723 (2)	168 (2)
N2—H2*F*···O3*B*^ii^	0.95 (2)	2.50 (2)	3.187 (2)	129 (2)
N2—H2*F*···O4*B*^ii^	0.95 (2)	1.82 (2)	2.760 (2)	169 (2)
N1*A*—H1*E*···O3	0.96 (1)	2.59 (2)	3.279 (2)	129 (2)
N1*A*—H1*E*···O4	0.96 (1)	1.86 (2)	2.811 (2)	169 (2)
N1*A*—H1*F*···O1*C*^iii^	0.92 (2)	1.90 (2)	2.787 (2)	162 (2)
N2*A*—H2*G*···O4*A*	0.92 (2)	1.81 (2)	2.710 (2)	164 (2)
N2*A*—H2*H*···O1*B*^iv^	0.95 (2)	1.82 (2)	2.764 (2)	168 (2)
N2*A*—H2*H*···O2*B*^iv^	0.95 (2)	2.51 (2)	3.191 (2)	128 (2)
O2—H2···O3	1.15 (3)	1.28 (3)	2.4258 (19)	174 (2)
O2*A*—H2*I*···O3*A*	1.21 (2)	1.22 (2)	2.4240 (19)	175 (2)
O3*B*—H2*J*···O2*B*	1.18 (2)	1.24 (2)	2.4174 (18)	177 (2)
O2*C*—H2*K*···O3*C*	1.20 (2)	1.22 (2)	2.4192 (19)	174 (2)

Symmetry codes: (i) *x*, *y*−1, *z*; (ii) *x*−1, *y*, *z*; (iii) *x*+1, *y*, *z*; (iv) *x*, *y*+1, *z*.
