# Supplementary figures and images for: Crystal structure of 2-methyl­piperazine-1,4-diium bis­(hydrogen maleate)
Source: Acta Crystallogr E Crystallogr Commun. 2015 Feb 21;71(Pt 3):o193–4. doi: 10.1107/S2056989015003102 (PMC4350694; doi:10.1107/S2056989015003102)

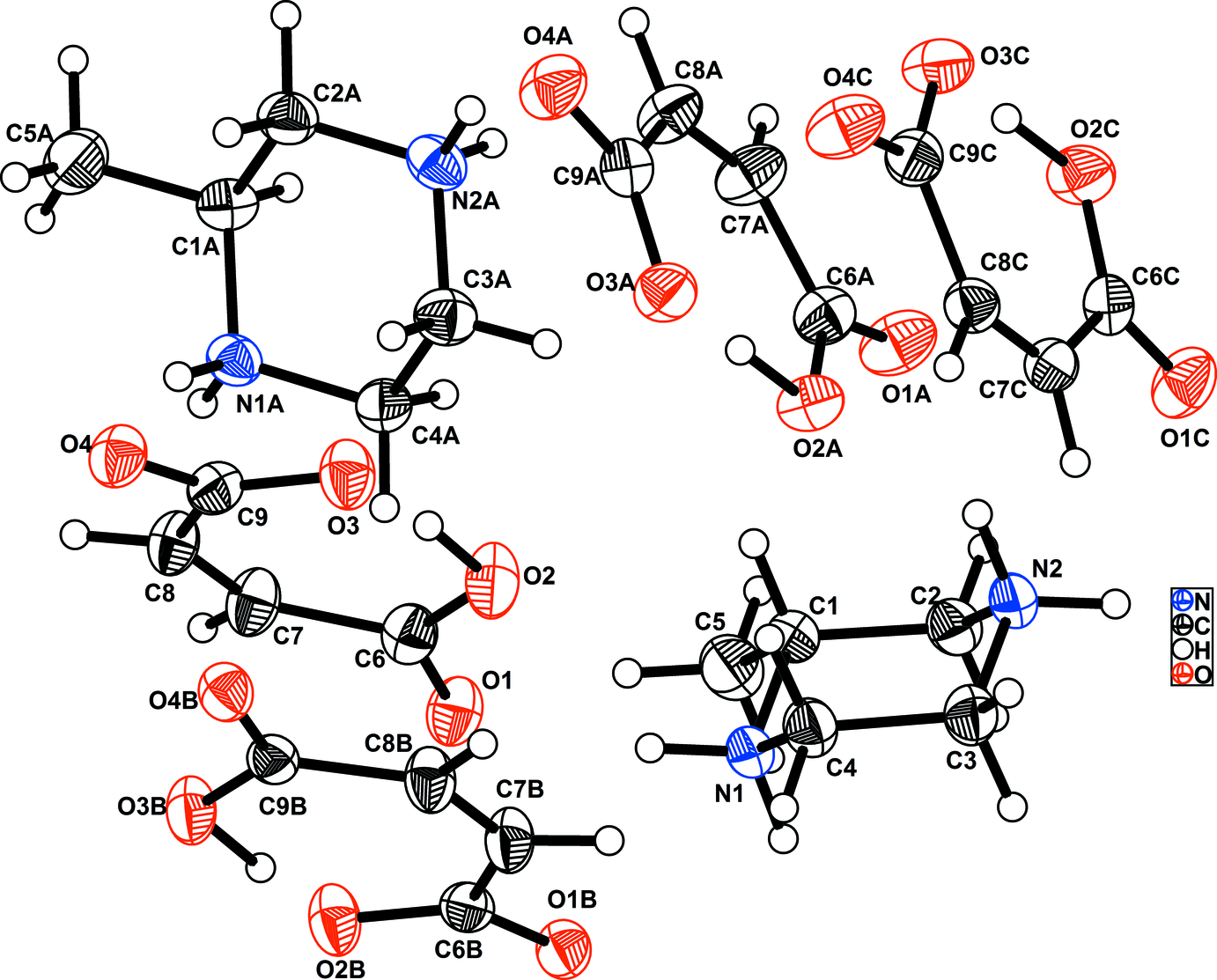

Supplement: Supplementary file 4 [file e-71-0o193-fig1.tif]

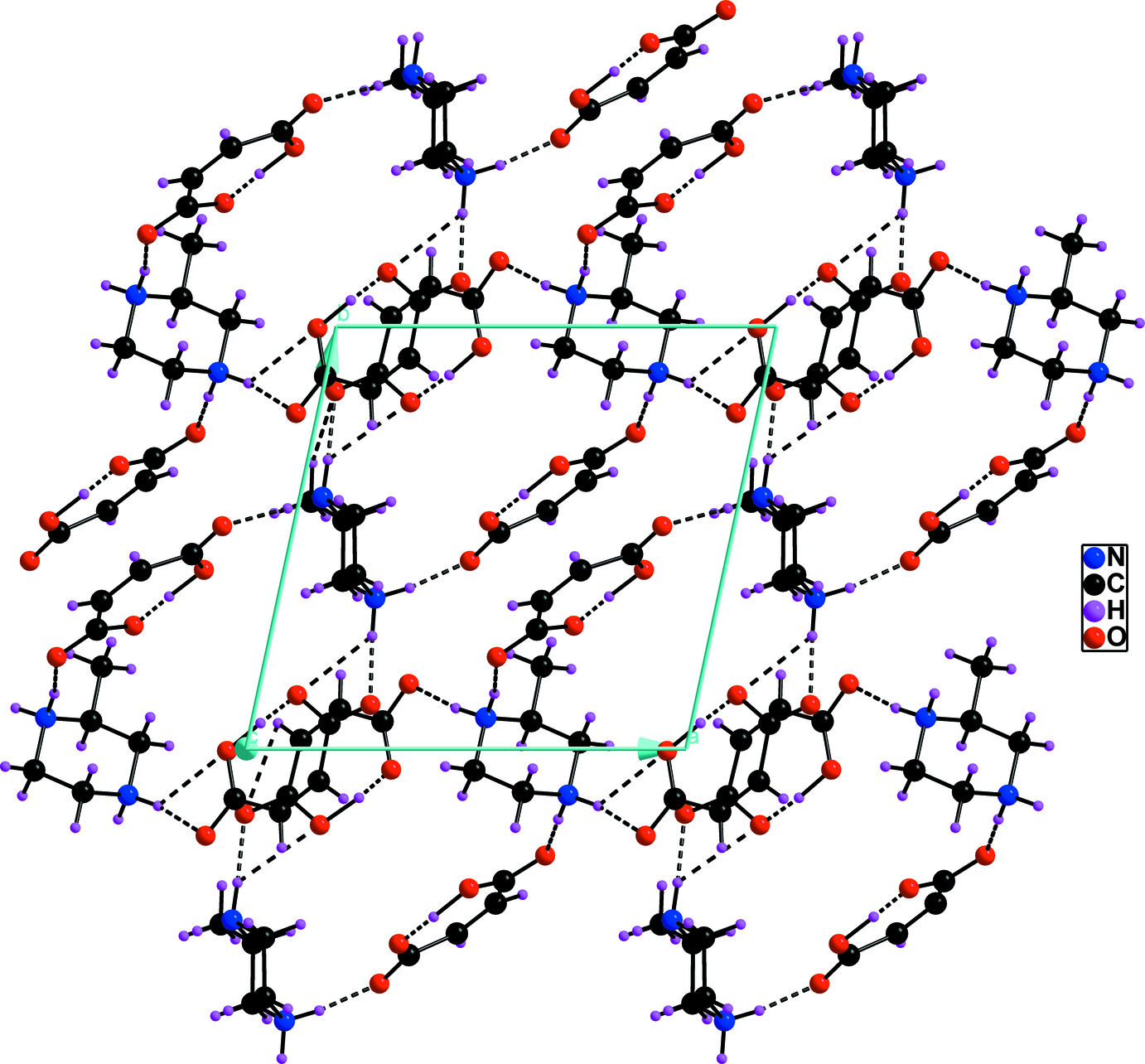

Supplement: Supplementary file 5 [file e-71-0o193-fig2.tif]

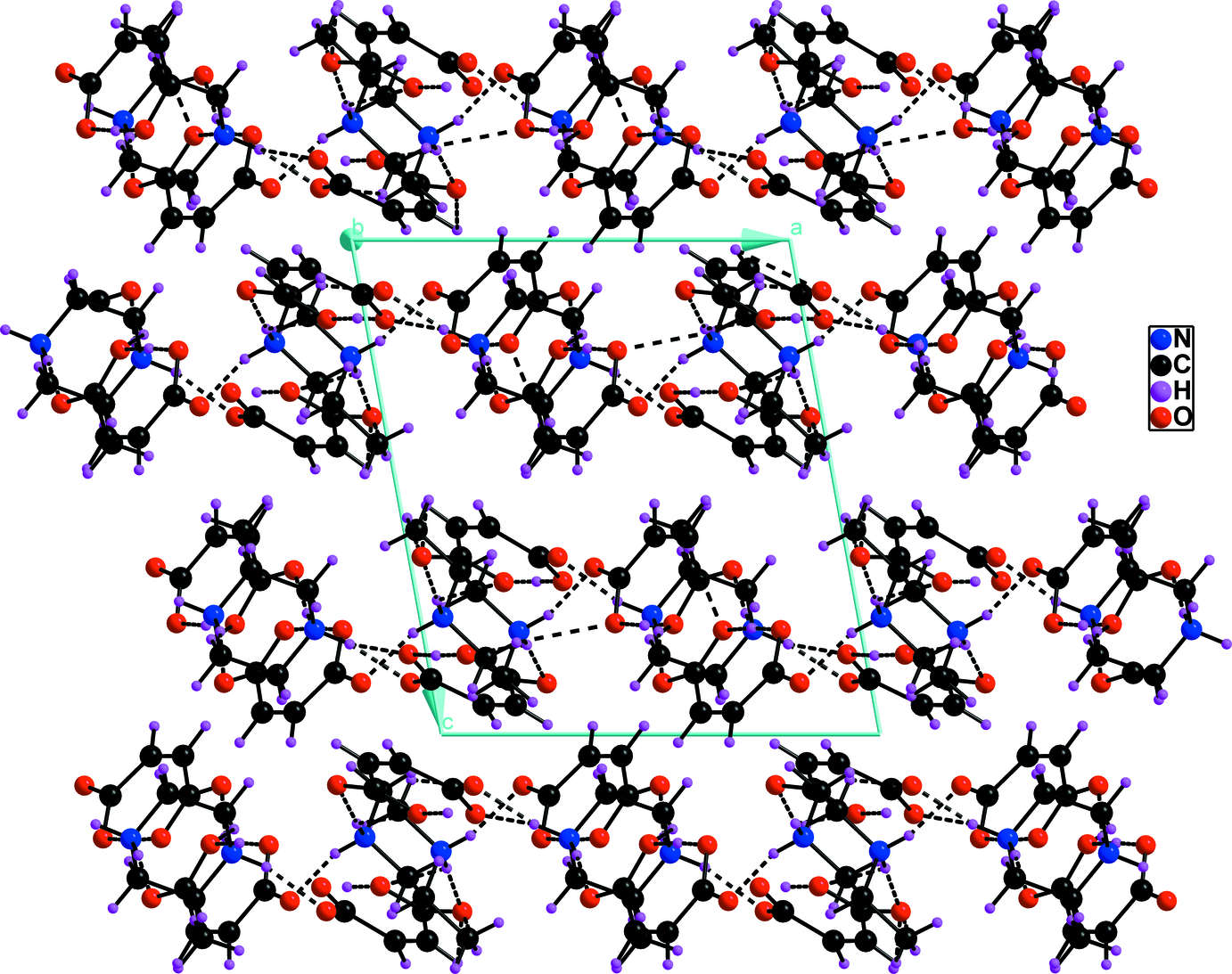

Supplement: Supplementary file 6 [file e-71-0o193-fig3.tif]
